# The poor prognosis of sarcomatoid carcinoma arising from low grade serous ovarian cancer: A case report and review of the literature

**DOI:** 10.1016/j.gore.2021.100735

**Published:** 2021-02-23

**Authors:** Paulina Haight, Johanna Savage, Kristin Bixel

**Affiliations:** a Division of Gynecologic Oncology, The Ohio State University Wexner Medical Center, M210 Starling Loving Hall, 320 W 10^th^ Avenue, Columbus, OH 43210, United States; b Division of Pathology, The Ohio State University Wexner Medical Center, S305E Rhodes Hall, 410 W 10^th^ Avenue, Columbus, OH 43210, United States

**Keywords:** Low grade serous carcinoma, Serous borderline tumor, Atypical proliferative serous tumor, Sarcomatoid carcinoma, Anaplastic carcinoma

## Abstract

Sarcomatoid or anaplastic carcinomas arising within epithelial ovarian neoplasms are rare, particularly within serous tumors of the ovary, and carry a poor prognosis. Here we present the case of a patient initially diagnosed with low grade serous ovarian carcinoma arising within a serous borderline tumor (atypical proliferative serous tumor). She underwent rapid progression of disease and was found to have anaplastic/sarcomatoid carcinoma on biopsy of recurrence. These findings prompted pathologic re-review of the original neoplasm, which demonstrated a microscopic focus of sarcomatoid/anaplastic carcinoma.

## Highlights

• Sarcomatoid or anaplastic carcinomas arising within serous ovarian neoplasms are rare.
• Despite small size, sarcomatoid carcinomas are aggressive spindle cell nodules.
• Clinician communication with the pathologist is critical when inconsistency exists.

## 1 Introduction

Epithelial tumors of the ovary are commonly pure, but a subset may demonstrate sarcomatous elements or anaplastic foci. The most common biphasic tumor is carcinosarcoma (malignant mixed Mullerian tumor). However, “mural nodules” have been described as arising within cystic epithelial tumors of the ovary in rare circumstances. These nodules can be classified as either reactive proliferations (“sarcoma-like” mural nodules), true sarcomas or sarcomatoid/anaplastic carcinomas (De Rosa et al., 1991). Distinguishing the former benign lesion from the latter two malignant lesions is of clinical importance for therapy and prognosis (Prat and Scully, 1979). Unfortunately, when a mural nodule is not grossly apparent, identification of true sarcomas or sarcomatoid/anaplastic carcinoma can be challenging and is subject to sampling error.

Most cases of malignant spindle cell mural nodules have been reported in association with mucinous ovarian tumors, with the first being described by Prat and Scully in 1979 (Prat and Scully, 1979, Prat and Scully, 1979). The finding of similar nodules in ovarian serous neoplasms has been rarely described, with limited case reports published in the literature (De Rosa et al., 1991, Clarke, 1987, McCullough et al., 1988, Andrews et al., 2008, Garg et al., 2012). Four of these describe sarcomatoid or anaplastic carcinomas arising in low-grade serous neoplasms (De Rosa et al., 1991, Clarke, 1987, McCullough et al., 1988, Andrews et al., 2008). Interestingly, Garg et al describe two cases of serous borderline tumors which demonstrated high-grade transformation to sarcomatoid/anaplastic carcinoma at the time of recurrence (Garg et al., 2012). In this article we present a case of sarcomatoid/anaplastic carcinoma arising within a low-grade serous ovarian neoplasm, provide a review of the current literature, and discuss management of these rare tumors.

## 2 Case report

The patient is a 57-year-old multiparous woman who presented to her primary care physician with complaints of abdominal pain and bloating. Imaging demonstrated an 8 cm complex adnexal mass with omental thickening, peritoneal carcinomatosis and ascites. CA-125 was elevated (928). She underwent an optimal primary tumor reductive surgery including total abdominal hysterectomy, bilateral salpingo-oophorectomy, pelvic lymph node dissection, omentectomy, and Argon laser ablation of tumor deposits on the mesentery. Intra-operatively, she was found to have tumor involving bilateral tubes and ovaries, the mesentery of the rectosigmoid, epiploica and omentum. Intra-operative frozen section was consistent with serous carcinoma. Final pathology demonstrated low-grade serous carcinoma of the ovary, arising from a serous borderline tumor, FIGO stage IIIC.

The patient was offered standard of care adjuvant chemotherapy followed by hormone therapy maintenance, versus participation in clinical trial comparing this regimen to hormone therapy. She elected to proceed on clinical trial (GY-019) and was randomized to a regimen of letrozole 2.5 mg daily for 6 cycles, followed by letrozole maintenance. Her post-operative CA-125 declined to 52. A CT scan prior to initiating therapy showed ill-defined nonspecific peritoneal thickening in the pelvis.

**Fig. 1:**
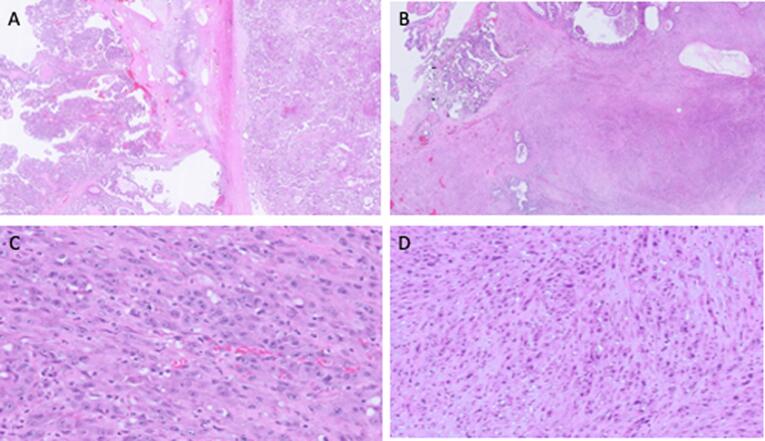
Histology of low-grade serous tumor with focus of anaplastic carcinoma. Legend. (A) Initial ovarian mass, with areas morphologically consistent with borderline serous tumor and invasive low-grade serous carcinoma (10× magnification); (B-C) re-review of the original pathology revealed borderline serous tumor and anaplastic carcinoma (10× magnification and 400× magnification, respectively); (D) sarcomatoid/anaplastic carcinoma from the peritoneal recurrence, demonstrating similar morphologic features (400× magnification).

Less than two months following the primary tumor reductive surgery (and one week after starting adjuvant letrozole therapy), the patient presented with a sepsis-like picture including fever, abdominal pain and distention, and leukocytosis. Repeat imaging showed ascites and progression of peritoneal nodules in the pelvis. She was started on broad-spectrum antibiotics however her clinical condition deteriorated. Physical examination was concerning for peritonitis and she was taken to the OR for exploratory laparotomy. Intraoperative findings included loculated, non-purulent ascites and edematous bowel without evidence of perforation. Microbial cultures of the ascites fluid were negative. Biopsies of soft tissue nodules along the mesentery and small bowel nodule revealed a high-grade malignant neoplasm with epithelioid and spindle cell morphology. The tumor cells were weakly positive for pancytokeratin (AE1/AE3) and strongly positive for CAM5.2. Patchy staining with desmin and SMA were also observed. The combined morphologic and immunohistochemical findings were consistent with a sarcomatoid/anaplastic carcinoma. This prompted review of her original pathology, and a microscopic focus of sarcomatoid/anaplastic carcinoma was identified in the right ovary (Fig. 1).

She was started on chemotherapy (carboplatin and paclitaxel) and initially demonstrated dramatic clinical improvement. Unfortunately, she was diagnosed with progressive disease after 4 cycles of chemotherapy and died of disease 7 months after her initial diagnosis.

## 3 Discussion

The first “sarcoma-like nodules” were reported within mucinous ovarian tumors by Prat and Scully in 1979. In their reports, these nodules either had a “sarcoma-like” appearance (well-circumscribed nodules with multinucleated giant cells) or a malignant/sarcomatous appearance. The “sarcoma-like” nodules tended to have a latent clinical course, whereas the true sarcomatous and malignant nodules behaved in aggressive fashion, resulting in patient death within 1.5 years of diagnosis (Prat and Scully, 1979, Prat and Scully, 1979). Since that time, several additional case reports have described similar sarcomatoid or anaplastic nodules arising within mucinous ovarian neoplasms (Chan et al., 1989, Chang et al., 2005, Tsuruchi et al., 1993).

Distinction between benign “sarcoma-like” and malignant true sarcomatous or sarcomatoid/anaplastic carcinoma can be difficult. “Sarcoma-like” nodules are typically small and well-demarcated. They typically exhibit one of three patterns: pleomorphic and epulis-like (sheets of multinucleated giant cells with scattered mononuclear cells), pleomorphic and spindle cell (spindled cells with hyperchromatic nuclei, a prominent inflammatory infiltrate, and scattered multinucleated giant cells), and giant cell histiocytic (sheets of mononuclear cells with abundant cytoplasm and low mitotic activity). True sarcomatous nodules and sarcomatoid/anaplastic carcinomatous foci are typically ill-defined. Sarcomatous nodules exhibit morphologic and immunohistochemical profile in keeping with the malignant cell type. Sarcomatoid/anaplastic carcinomas may have more variable morphology, with both epithelioid and spindle cell. Cytokeratin stains are typically diffuse and strongly positive in sarcomatoid/anaplastic carcinoma but may be focal (Andrews et al., 2008).

Sarcomatoid or anaplastic carcinoma has rarely been reported in serous ovarian neoplasms (Table 1). Clarke described the first case of an anaplastic carcinoma arising in a serous borderline tumor in 1987 (Clarke, 1987). Since that time, three additional reports have been published describing mural nodules of anaplastic or sarcomatoid carcinoma arising within borderline or low-grade serous ovarian neoplasms. In the reported cases, all patients underwent tumor reductive surgery. Regardless of adjuvant therapy, clinical outcomes were generally poor. One out of 7 patients is alive, with only 6 months of documented follow-up. The remainder of patients have died of disease, with most patients unfortunately succumbing within 6–8 months of sarcomatoid/anaplastic carcinoma diagnosis. The longest documented survival is 32 months after initial diagnosis (De Rosa et al., 1991, Clarke, 1987, McCullough et al., 1988, Andrews et al., 2008).

**Table 1:** Summary of case reports of sarcomatoid carcinoma arising in serous ovarian cancer.

Author	Patient age at initial diagnosis	Initial diagnosis	Gross description of tumor	Histologic features of mural nodule	Adjuvant therapy	Time to recurrence	Clinical outcome
DeRosa	41	Serous borderline, multiple mural nodules of sarcomatoid carcinoma	20 × 17 × 15 cm multiloculated serous cystic tumor with multiple fleshy nodules	Spindle-shaped hyperchromatic nuclei, high mitotic index, few entrapped benign glands, benign epithelium	No	3 months	Dead of disease 6 months after initial diagnosis
Clarke	72	Serous borderline with small foci of superficial, invasive papillary serous adenocarcinoma, one anaplastic / sarcomatoid nodule	22 cm unilocular cystic tumor with multiple yellow-tan mural nodules up to 2.5 cm in diameter	Undifferentiated spindle cells, IHC positive for vimentin and negative for cytokeratin	No	N/a	Dead of disease 8 months after initial diagnosis
Andrews	49	Serous borderline, one nodule of sarcomatoid carcinoma	8.5 × 7.0 × 5.5 cm multiloculated serous cystic tumor, with focal superficial papillary areas and a 2 cm nodule	Spindle cells in fascicular pattern in addition to epithelioid and rhabdoid cells, focal necrosis, IHC positive for vimentin and CK7	Not initially; but did receive chemotherapy after recurrence	8 months	Dead of disease 32 months after initial diagnosis
McCullough	44	Bilateral serous cystadeno-carcinoma, one unilateral nodule of sarcomatoid carcinoma	23 cm ovarian cyst	Sarcomatous differentiation with rhabdomyoblasts and liposarcoma, IHC positive for desmin	N/a	N/a	Alive at 6 months after initial diagnosis, otherwise unknown
Garg	22 (borderline)	Bilateral serous borderline	N/a	N/a	Multiple chemotherapy cycles including Doxorubicin, Bevacizumab, Paclitaxel	10 years (multiple prior borderline recurrences); final recurrence sarcomatoid carcinoma without low-grade component	Dead of disease one month after high-grade recurrence
	47 (borderline)	Bilateral serous borderline	N/a	N/a	N/a	3 years; recurred as sarcomatoid carcinoma without low-grade components	Dead of disease 6 months after high-grade recurrence
Haight	57	Low grade serous, microscopic focus of anaplastic / sarcomatoid carcinoma	13.3 cm serous cystic tumor with excrescences	Epithelioid and spindle cell morphology, IHC positive for keratin	Initially Letrozole, then Carboplatin, Paclitaxel	2 months	Dead of disease 7 months after initial diagnosis

Interestingly, in 2012 Garg reported a series of three patients who presented with high-grade transformation of their ovarian neoplasms at the time of recurrence (Garg et al., 2012). These patients were initially diagnosed with low-grade serous neoplasms (two patients with bilateral serous borderline tumors, and one patient with low-grade serous carcinoma). The high-grade transformation was classified as sarcomatoid/anaplastic carcinoma in two patients, and as a true carcinosarcoma in the other patient. Original pathology was re-reviewed in each case and did not demonstrate evidence of a malignant mural nodule. Both patients with sarcomatoid/anaplastic carcinoma died of disease shortly after high grade recurrence.

The expected clinical course of a borderline or low-grade neoplasm is indolent. Primary therapy consists of surgical resection. The optimal adjuvant therapy for low grade serous carcinoma is currently being investigated in GY019 as above, to which this patient was enrolled in the hormone therapy alone arm. While rare, the presence of a sarcomatoid/anaplastic carcinoma component within a borderline or low-grade neoplasm appears to dramatically alter the behavior of the tumor. Patients and providers can expect a more aggressive clinical course, with most reports demonstrating poor prognosis. In keeping with prior reports, our patient underwent rapid disease progression despite the malignant mural nodule comprising a very small proportion of her original tumor that was not grossly visualized.

From our experience, communication between the clinician and pathologist is critical when the clinical course does not correlate with initial pathologic diagnosis. In our case, the patient’s rapid progression was discordant with original diagnosis of low-grade neoplasm, which prompted re-review of the initial specimen. While re-review may not always resolve the discrepancy, it may demonstrate previously unappreciated foci of sarcomatoid/anaplastic carcinoma and alter treatment plan. In our case, the patient was promptly started on adjuvant chemotherapy, but her disease ultimately progressed despite initial clinical improvement.

In summary, sarcomatoid/anaplastic carcinoma of the ovary can develop in rare cases as a mural nodule within borderline or low-grade serous ovarian neoplasms. Although characterized by grossly favorable features, most patients undergo rapid disease progression and poor outcome. It is important to distinguish between a benign “sarcoma-like” mural nodules and malignant true sarcomas or malignant sarcomatoid/anaplastic carcinomas. Specifically, we advocate that the presence of a sarcomatoid/anaplastic carcinoma nodule should prompt treatment with adjuvant chemotherapy, as the clinical course tends to mimic high-grade ovarian cancer despite a large component of the tumor being borderline or low grade.

## 4 Conclusion

Despite their association with borderline or low-grade cystic ovarian neoplasms, most reports of sarcomatoid or anaplastic carcinoma describe aggressive clinical behavior. We agree with the arguments of prior authors who advocate that these lesions should be classified distinctly and treated in the same manner as high-grade ovarian carcinomas despite otherwise favorable features.

## Informed consent

Verbal informed consent was obtained from the patient’s next of kin for publication of this case report on 12/14/2020. A copy of the written consent is available for review by the Editor-in-Chief of this journal on request.

## CRediT authorship contribution statement

**Paulina Haight:** Conceptualization, Data curation, Formal analysis, Investigation, Writing - original draft. **Johanna Savage:** Data curation, Visualization, Writing - review & editing. **Kristin Bixel:** Conceptualization, Formal analysis, Supervision, Writing - review & editing.

## Declaration of Competing Interest

The authors declare that they have no known competing financial interests or personal relationships that could have appeared to influence the work reported in this paper.
